# Thin films of Type 1 collagen for cell by cell analysis of morphology and tenascin-C promoter activity

**DOI:** 10.1186/1472-6750-6-14

**Published:** 2006-03-06

**Authors:** Kurt J Langenbach, John T Elliott, Alex Tona, Dennis McDaniel, Anne L Plant

**Affiliations:** 1 Biotechnology Division/National Institute of Standards and Technology, Gaithersburg, MD 20899, USA; 2 Geo-centers, Inc. Newton, MA 02459, USA

## Abstract

**Background:**

The use of highly reproducible and spatiallyhomogeneous thin film matrices permits automated microscopy and quantitative determination of the response of hundreds of cells in a population. Using thin films of extracellular matrix proteins, we have quantified, on a cell-by-cell basis, phenotypic parameters of cells on different extracellular matrices. We have quantitatively examined the relationship between fibroblast morphology and activation of the promoter for the extracellular matrix protein tenascin-C using a tenascin-C promoter-based GFP reporter construct.

**Results:**

We find that when considering the average response from the population of cells, cell area correlates with tenascin-C promoter activity as has been previously suggested; however cell-by-cell analysis suggests that cell area and promoter activity are not tightly correlated within individual cells.

**Conclusion:**

This study demonstrates how quantitative cell-by-cell analysis, facilitated by the use of thin films of extracellular matrix proteins, can provide insight into the relationship between phenotypic parameters.

## Background

Extracellular matrix (ECM) proteins can induce diverse intracellular signals by providing both mechanical and chemical stimuli to cells. These intracellular signals will mitigate and be affected by other sources of cell signals. Because of the importance of ECM cues in signaling pathways, careful control of the ECM is extremely important when studying downstream signaling events or cellular response to soluble factors such as pharmaceuticals. In this study, we demonstrate the use of highly homogeneous and reproducible thin films of collagen at two different concentrations, and thin films of fibronectin, to provide different extracellular matrix signals to mouse embryonic fibroblasts (NIH3T3) cells. The high level of homogeneity of thin films affords the possibility that each cell in a population has the same microenvironment and therefore each has very similar stimuli.

We report here the quantification of cellular parameters, including intracellular green fluorescent protein (GFP) fluorescence intensity, on a cell by cell basis, for large numbers of adhered cells. Thin films facilitate the collection of unbiased and quantitative data from many fields of cells in an automated fashion. Because the films are very homogeneous in thickness, autofocusing is more reliable; and because they are optically thin, background signal from scattered light is minimal.

We have directly examined the correlation between the spreading of cells and tenascin-C (TN-C) promoter activity in cells transfected with a GFP-TN-C promoter construct. It has been frequently reported that cell morphology and cytoskeletal arrangement appear to influence integrin-dependent induction of gene expression [1-4]. Expression of the extracellular matrix protein, TN-C, is an example of an apparent linkage between gene expression and cell morphology. TN-C expression is regulated by a number of mechanisms including growth factors, biomechanical force and ECM proteins, and discrete sites on the promoter region control these responses [5-7]. TN-C expression in fibroblasts has been shown to be associated with adhesion and spreading on fibronectin, and is differentially responsive to various collagen preparations [8-10]. Surfaces that support enhanced cell spreading (fibronectin and monomeric collagen) have been reported to induce increased TN-C promoter activity [8-11]. Furthermore, an intact cytoskeleton appears to be required for TN-C expression as disruption of the actin cytoskeleton with cytochalasin D results in a reduction of TN-C protein production in the population, even when cells can still actively engage αvβ3 integrin [9,12].

Despite such observations suggesting a link between cell morphology and gene expression, the precise mechanism by which this influence occurs remains obscure. If the pathways that determine cell morphology also influence TN-C gene expression, we might expect a strong correlation between the two cellular parameters in each cell. To carry out such an analysis requires assurance that the extracellular matrix presented to cells is homogeneous and reproducible, so that all cells are receiving the same signals across a sample and between replicate samples. These criteria cannot be assured for collagen gels, which are difficult to characterize with regard to structure and homogeneity, and are often fragile and difficult to handle. Such an analysis also requires quantitative analysis of a statistically significant number of adhered cells. Thus we employed thin films of collagen and fibronectin, which provide the homogeneity, reproducibility, and desired optical properties.

Thin films of the ECM proteins were formed by allowing collagen and fibronectin molecules from solution to adsorb to a hydrophobic alkanethiol surface. Thin films of fibronectin on alkanethiol have been fabricated and characterized by others [13-17]. We have previously reported the preparation of thin films of Type 1 collagen, along with extensive analysis of these films by atomic force microscopy, ellipsometry and light microscopy [18]. Type 1 collagen readily forms large fibrils on the alkanethiol surface in the presence of collagen in solution at concentrations >300 μg/mL. At this concentration of collagen the fibrils formed are predominately 200 nm – 250 nm in diameter and at least several microns in length, similar to the dimensions of triple-helical collagen fibrils as observed with electron microscopy [19]. We have demonstrated by comparison of a number of parameters that A-10 vascular smooth muscle cells respond similarly on thin films of collagen as they do on thick hydrogels of collagen [18].

In this study, we use thin films of collagen and fibronectin to collect image data on large numbers of NIH3T3 fibroblasts to quantify the occurrence of multiple parameters in individual cells. Cell area, cytoskeletal organization, and TN-C promoter activity as indicated by GFP expression, are quantitatively determined on a cell-by-cell basis by fluorescence microscopy. These data show that even clonal populations on spatially homogeneous thin films show a range of responses. Although fibroblasts on any one of the homogeneous matrices show a distribution of responses across the population, the average response and the range of responses are distinct according to which of the different matrices are used. We have observed on a cell-by-cell basis that the amount of cytoskeletal staining with phalloidin in individual cells is correlated with their cellular area. Examination of cell area and TN-C promoter activity as indicated by GFP intensity shows that on the average, cell area and average GFP intensity appear to be correlated, but cell-by-cell analysis indicates at best a very weak correlation between cell spreading and promoter activity within individual cells.

## Results

### Fibroblast morphology on thin films of extracellular matrix proteins

**Figure 1:**
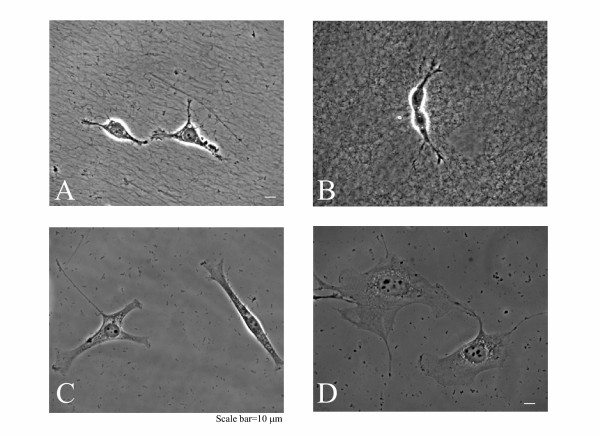
Phase contrast images of NIH3T3 cells on (A) a thin film of fibrillar collagen, (B) a gel of fibrillar collagen, (C) a thin film prepared from a lower concentration of collagen, (D) a thin film of fibronectin. Microscopy images were taken using a 40× objective and the calibration bar equals 10 μm. The small particles observed on the surface are a result of fixative precipitation during the fixing protocol used to preserve the cytoplasmic GFP protein within the cell.

Representative images of NIH3T3 cells grown on three different collagen matrices, one hydrogel and two thin films, and a thin film of fibronectin are shown in Figure 1. In Figure 1A and 1B, the high density of thick collagen fibrils is readily apparent by light microscopy. The fibrillar collagen in Figure 1B is in the form of a thick (≈0.4 mm – 1 mm) hydrogel, and is a type of collagen preparation that has been frequently utilized as a model for *in vivo *Type 1 collagen [20,21]. Figure 1A depicts collagen fibrils of similar dimension as in Figure 1B, but in the form of a very thin film of tens of nanometers in thickness, which is formed by the polymerization of collagen from solution (0.3 mg/mL collagen) at a surface coated with an alkanethiol monolayer.

The thin film prepared from the lower concentration of collagen (≈0.025 mg/mL w/v) is shown in Figure 1C. This solution concentration of collagen is often used for coating polystyrene and glass coverslips. We have previously demonstrated by atomic force microscopy [18] that films of collagen formed from a low solution concentration of collagen consist primarily of very small fibrils (<50 nm in diameter and approximately 250 nm in length, which are too small to be seen by light microscopy) and a very low density of the larger fibrils that characterize the thin films prepared from higher concentrations (> 0.3 mg/mL w/v). The thin film shown in Figure 1D is prepared by adsorbing fibronectin to an alkanethiol monolayer. Thin films of fibronectin have been used in a number of previous studies [13,14,16,22].

As can be seen by comparing Figure 1A and 1B with Figure 1C and 1D, cells grown on a matrix of dense fibrillar collagen, whether as a thin film or a hydrogel (Figure 1A and 1B) are more compact than cells grown on the thin films of fibronectin (Figure 1D) or thin films prepared from lower concentrations of collagen (Figure 1C). The cells on the dense fibrillar collagen tend to form clusters and exhibit few but more prominent pseudopod-like extensions. These cells assume a stellate or bipolar morphology as previously described [23-26]. Fibroblasts cultured on thin films of fibronectin (Figure 1D) show a well-spread morphology consistent with previous studies [13-16]. Fibroblasts seeded onto the thin films prepared from the lower concentration of collagen, which have a low density of large fibrils, display an intermediate morphology with small projections and they are more spread than cells on the substrate of dense collagen fibrils (Figure 1C).

### Quantitative analysis of cell morphology

**Figure 2:**
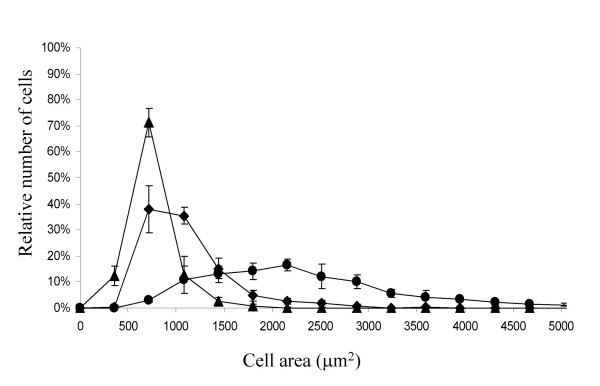
NIH3T3 cells transfected with the GFP-TN-C promoter based reporter construct were plated onto the different surface preparations at the same density, and after 24 hours they were fixed, stained with DAPI and Texas Red-C_2_-maleimide. For each surface, all the cells in 50 fields were analyzed using automated microscopy (with several hundreds of cells sampled per surface). Fibronectin thin film, ●; thin film of fibrillar collagen (▲), thin film prepared from a lower concentration of collagen (◆). Error bars reflect the standard deviation of a minimum of four samples derived from at least two replicate experiments. While these data are the results from transfected fibroblasts, untransfected NIH3T3 cells exhibit equivalent areas and distributions of areas in response to these different matrices.

The images shown in Figure 1 provide qualitative evidence of fibroblast morphology on these matrices. To quantify the morphological changes, cells were stained and examined using automated fluorescence microscopy and image analysis software [18,27]. The staining protocol used was developed to allow us to very accurately discriminate cell edges from the background. Figure 2 shows the distribution of cell area for the NIH3T3 fibroblasts containing the GFP-TN-C promoter based reporter construct that were cultured for 24 hours on each of the different thin film matrices. Analysis of several hundred cells on each surface reveals that on thin films of fibrillar collagen, most cells are less spread than most cells on the thin films prepared from the lower concentration of collagen, which are less spread than most cells on fibronectin. It is notable that each cell population, while distinct from the other populations in the extent of cell spreading, displays a range of cell areas. This observation indicates a large degree of heterogeneity in cellular morphology even though the population is clonal and the surfaces are highly homogeneous when cells are seeded onto them. The population distributions for cell area on each of the matrices are highly reproducible (Figure 2) suggesting that the distributions reflect inherent variability in cell response.

### Immunofluorescent detection of actin organization and associated proteins

To examine some of the signaling events that may connect morphological differences to promoter activation, we qualitatively evaluated the presence of integrin-related signaling proteins, and the presence of filamentous actin (F-actin) organization. Fibroblast adhesion to native collagen is primarily mediated via interactions with the integrins α2β1 and α1β1 [28,29], while several integrins including αVβ3 and α5β1 mediate binding to fibronectin [30-33]. Integrin ligation and clustering can lead to formation of focal adhesions, reorganization of the actin cytoskeleton and activation or modulation of intracellular signaling molecules depending upon the integrin involved [34-36]. Transcription factors that induce TN-C expression in response to changes in integrin engagement have been identified [8,37,38]. Jones et al. have demonstrated a role for the ERK (extracellular signal-regulated kinase) pathway and in more recent studies they showed that FAK (focal adhesion kinase) activation is required for TN-C expression [8,37]. Cells lacking FAK, or cells in which a dominant negative FAK is introduced, have lower levels of TN-C expression and deposition [8].

**Figure 3:**
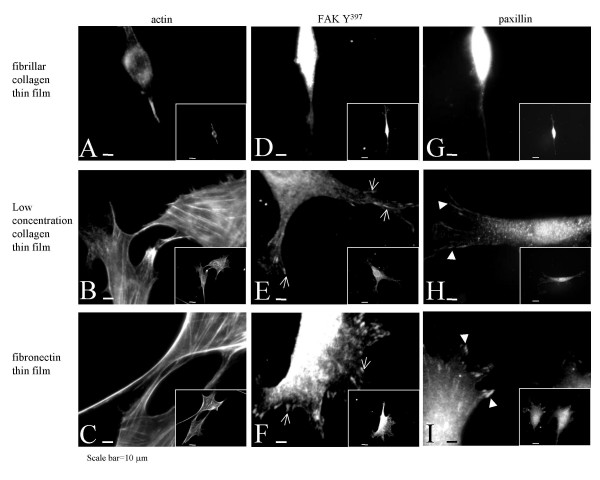
NIH3T3 cells were cultured on the different surfaces for 24 hours and then fixed for immunofluorescence staining. Panels A-C are cells labeled with FITC-phalloidin. Panels D-F are cells labeled with antibodies directed against FAK phosphorylated on tyrosine 397 (FAK Y^397^) and panels G-I are cell labeled with antibodies directed against paxillin. Preparations in Panels (A, D, G) are thin films of fibrillar collagen; (B, E, H) are thin films prepared from the lower concentration of collagen; and (C, F, I) thin films of fibronectin. Arrows in E, F and arrowheads in G, H identify FAK Y^397 ^staining and paxillin staining localized in focal adhesion-like plaques respectively. Microscopy images were taken using a 40× objective and the calibration bar equals 10 μm.

Figure 3 shows representative images of the cytoskeleton, the focal adhesion associated protein paxillin, and FAK phosphorylation staining in cells grown on the three different matrices. Cells grown on either the thin films prepared from the lower concentration of collagen or on the thin films of fibronectin had more extensive plaque-like paxillin staining localized primarily in the cell periphery consistent with the formation of classic focal adhesion complexes (arrowheads in panels H and I Figure 3), and well-delineated actin stress fibers stained with phalloidin (Panels B and C). In contrast, cells on the thin films of fibrillar collagen (Panel G) have few if any paxillin plaques. Instead, paxillin staining is found to be more diffuse perhaps associating with immature focal complexes [39]. Cells on the thin film of fibrillar collagen display very diffuse staining of phosphorylated FAK (FAK Y^397^) (Figure 3D) while cells on the thin films prepared from lower collagen concentrations or fibronectin show staining in focal adhesion-like plaques (arrows in Figure 3H and 3I). Phalloidin staining of the actin cytoskeleton in Figure 3 reveals little indication of stress fiber formation in cells growing on the thin film of fibrillar collagen (Figure 3A), especially compared to those on fibronectin and the thin films prepared from the lower concentration of collagen (Figure 3C and 3B respectively).

The representative images shown in Figure 3 suggest that there is an increase in the number of paxillin- and phosphorylated FAK Y^397^-containing focal adhesions in cells that are better spread. The extent of spreading is reduced on the thin films of collagen fibrils, and the formation of dense plaques of paxillin and phosphorylated FAK Y^397 ^staining associated with mature focal adhesions is reduced. These data are consistent with recent reports showing a relationship between cell spreading and the formation of focal adhesions in endothelial cells [40,41]. The data in Figure 3 also show that cells on the thin films of fibrillar collagen do not develop actin stress fibers. These results are in good agreement with previous studies of fibroblasts on fibrillar collagen preparations showing that the cells typically display minimal actin cytoskeletal stress fibers, a loss of vinculin and talin localization in focal contacts, and a redistribution of tyrosine phosphorylation and α2 and β1 integrin subunits from focal adhesions [24].

### Quantitative analysis of tenascin-C promoter activity

The expression of the extracellular matrix protein TN-C has been previously linked to extent of cell spreading in response to collagen and to fibronectin preparations [5-9]. Cells that were able to actively spread and form focal adhesions or to generate sufficient tension showed increased levels of TN-C promoter activity as measured using promoter-based reporter constructs, semi-quantitative RT-PCR and microarrays [8-10].

**Figure 4:**
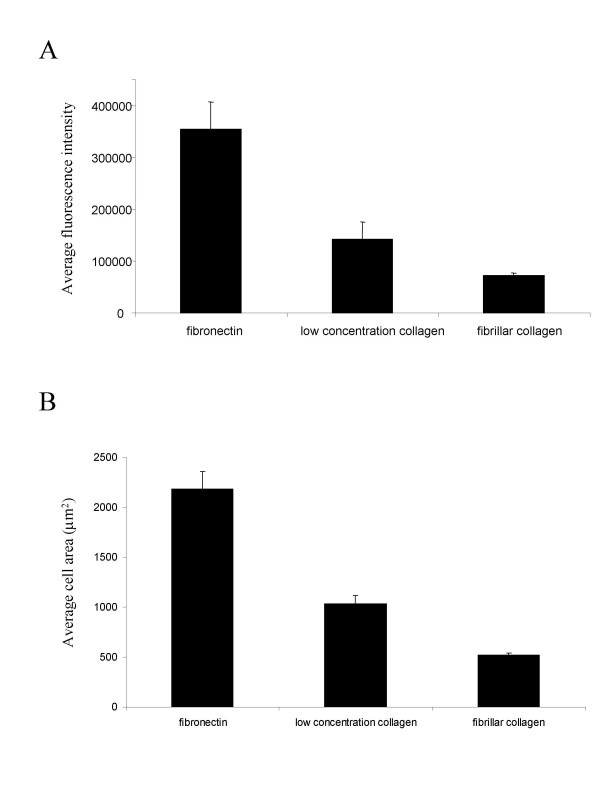
NIH3T3 cells transfected with the GFP-TN-C promoter construct were plated onto the different preparations at the same density, and at 24 hours after plating, they were fixed and stained with DAPI and Texas Red-C_2_-maleimide. Cells in 50 fields were imaged and analyzed on each sample using automated digital microscopy and image analysis software. A) Average relative GFP intensity is reported as a function of surface preparation. All data are statistically different from one another at p < 0.05. B) Average cell area is reported as a function of surface preparation. Error bars reflect the standard deviation of a minimum of four samples derived from at least two replicate experiments.

**Figure 5:**
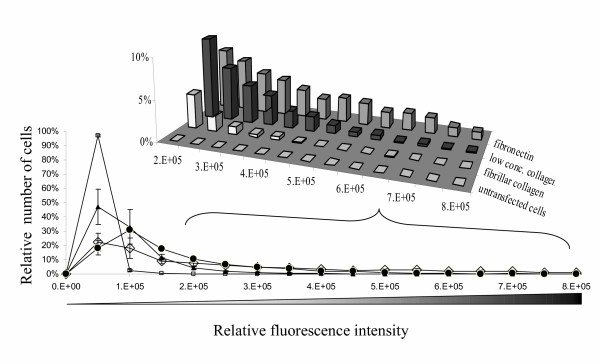
NIH3T3 cells transfected with the GFP-TN-C promoter construct were plated onto the different surfaces at the same density, and at 24 hours after plating they were fixed, stained with DAPI and Texas Red-C_2_-maleimide. Cells in 50 fields were imaged and analyzed on each sample using automated digital microscopy and image analysis software. Relative GFP fluorescence intensity is plotted versus relative cell number and plots show data from at least two independent experiments each consisting of duplicate samples. Thin films of fibronectin (◇); thin films of fibrillar collagen (▲); and thin films prepared from the lower concentration collagen (●). Error bars reflect the standard deviation of a minimum of four samples derived from at least two replicate experiments. Control cells (□) were not transfected with the GFP construct, and so their intensity reflects autofluorescence. **Inset**: A 3-dimensional rendering of the cellular GFP fluorescence intensity data ≥ 200,000 fluorescence units.

To measure TN-C promoter activity, we generated a green fluorescent protein (GFP) reporter construct consisting of a 4.1 kilobase fragment of the TN-C promoter driving a destabilized GFP gene, and derived single cell clonal populations of stably transfected fibroblasts containing this construct. To quantify GFP in individual cells, cells were fixed using a procedure that retains cellular GFP (See Methods section), stained by the procedure used to quantify cell shape, and the image mask that corresponds to each cell's shape was then used to delineate the area under which GFP fluorescence was integrated. Thus, this protocol allows us to measure the intensity of GFP within each cell as defined by the mask that is used for the determination of the morphology of that cell [27]. This protocol allows us to examine even the cells that are not expressing GFP. The average GFP intensity of all the cells on each of the different matrices is shown in Figure 4A. In addition, the intensity of each cell is plotted as part of a histogram representation (Figure 5). Examination of hundreds of cells on each matrix results in reproducible distributions of GFP intensity, analogous to the distributions seen for cell area shown in Figure 2.

The data displayed in Figure 4A indicates the average activity of the GFP- TN-C-promoter in cells on the three ECM matrices. In cells on fibronectin, the mean intensity of GFP is more than twice that of cells on the thin films prepared from the lower concentration of collagen and more than three times that of cells on the thin films of fibrillar collagen. Further, the data displayed in Figure 4B show that the average area of cells on fibronectin is about twice that of cells on thin films prepared from the lower concentration of collagen, and about three times that of cells on thin films of fibrillar collagen. Over all, these data agree with previous studies suggesting that GFP- TN-C-promoter activity is correlated with cell spreading. The data seem to indicate that cells that are more spread appear to have up-regulated TN-C promoter activity.

The population distribution of GFP-TN-C-promoter activity on the different matrices is shown in Figure 5. Non-transfected control cells are also shown. The intensity of the control cells is due to autofluorescence. Some or most of the cells on all three matrices had fluorescence that was equivalent to the level of autofluorescence (< 100,000 A.U.). The histogram bins that correspond to fibronectin show the presence of many cells with very high fluorescence intensity (> 400,000 A.U.) indicating that TN-C promoter activity is greatest on fibronectin films (Figure 5 inset). Cells grown on thin films prepared from the lower concentration of collagen also appear in the higher intensity bins, but fewer of them are in the highest bins, and more of them are in intermediate intensity bins (100,000 – 400,000 A.U.) compared to the cells on fibronectin. Cells on thin films of fibrillar collagen show a clustering of cells in the intermediate intensity bins, and also show that more of the cells remain at the level of autofluorescence compared to the cells on fibronectin or on the thin films prepared from the lower concentration of collagen. The relative fraction of cells that are near background autofluorescence levels (< 100,000 A.U.) is ≈50% on thin films of fibrillar collagen, ≈23% on thin films prepared from the lower concentration of collagen, and ≈19% on fibronectin. The distribution of GFP intensity for each population is also distinct from the other populations grown on the different matrices. The population of cells on fibronectin contains more high intensity cells (>400,000 A.U.) compared to the population on thin films prepared from the lower concentration of collagen, which in turn has more bright cells than the populations on thin films of fibrillar collagen (Figure 5 inset).

### Correlating cell area and tenascin-C promoter activity and cytoskeleton organization

**Figure 6:**
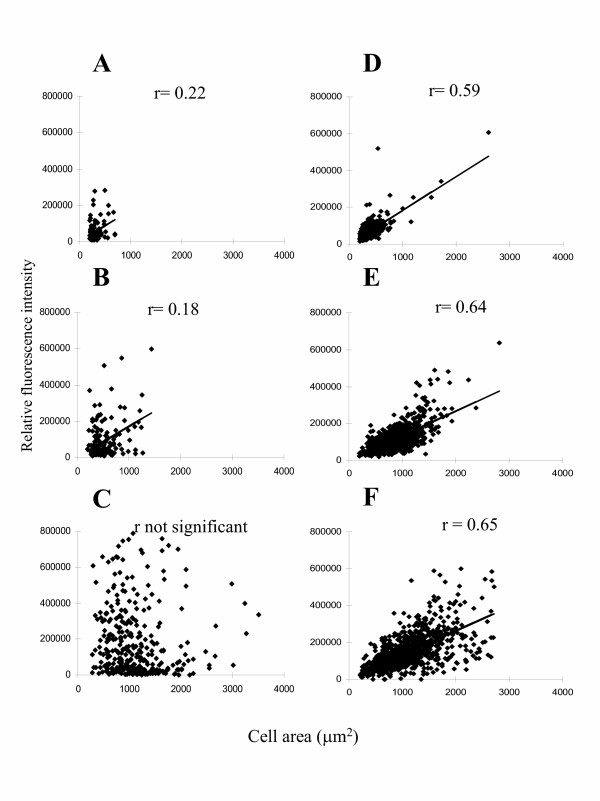
Cell-by-cell analysis of the relationship between cell area and GFP intensity or actin cytoskeleton in NIH3T3 cells. Panels A-C represent cells transfected with the GFP-TN-C promoter construct grown on (A) thin films of fibrillar collagen; (B) thin films prepared from a lower concentration of collagen; (C) thin films of fibronectin. Panels D-F represent FITC-phalloidin intensity in NIH3T3 cells on (D) thin films of fibrillar collagen; (E) thin films prepared from the lower concentration collagen; (F) thin films of fibronectin. Cells in 50 fields were imaged for each treatment. Lines are the result of least squares analysis. r = Spearman rank order correlation coefficient.

Although the mean GFP intensity and the mean cell area appear to be correlated on each of the substrates (Figure 4), we examined whether this correlation extends to the individual cellular level. If morphology and TN-C promoter activity were tightly correlated, we would expect that the larger cells would express more GFP. Figure 6 panels A-C show plots of cell area and GFP intensity on a cell-by-cell basis. Cells on the thin films of fibrillar collagen (panel A), thin films prepared from the lower concentration of collagen (panel B) and fibronectin (panel C) all fail to show a strong correlation between GFP intensity and cell area. The correlation coefficients (R values) of these plots as measured by the Spearman rank order correlation test are 0.22 and 0.18 with P values of 0.02 and 0.04, respectively for cells on the two collagen substrates, with no statistically significant correlation for the cells on fibronectin. A correlation coefficient of R = 1 indicates a perfect positive relationship between the two variables and the lower the P value the greater the probability that there is a direct relationship between the two variables. These data indicate a lack of a tight correlation between cell area and TN-C promoter activity.

As a test of our correlation analysis we examined the relationship between cytoskeleton organization and cell spreading. For this analysis, we used the same protocols for cell area determination, and the same microscopy and image analysis protocols, but examined nontransfected cells stained with fluorescently labeled phalloidin instead of examining them for GFP expression. These data are shown in Figure 6 panels D, E, F. Analysis using the Spearman rank order correlation test confirms that a strong correlation on a cell by cell basis exists between cell size and F-actin staining intensity. The measurement of the strength of the relationship between cell size and phalloidin staining resulted in a correlation coefficient of 0.59, 0.64 and 0.65 with P < 0.0001 for cells grown on thin films of fibrillar collagen, thin film prepared from the lower concentration of collagen, and fibronectin (Figure 6D, E and 6F) respectively. This indicates that the concentration of F-actin (i.e. stress fibers) within the cell is correlated with extent of cell spreading as has been previously suggested [42,43] whether the cells are on collagen or fibronectin matrices.

## Discussion

In this study, we examined large numbers of individual cells, and quantified the extent of cell spreading and the activity of a reporter construct composed of a fragment of the TN-C promoter driving a degenerate version of enhanced GFP. This variant of GFP is reported to have a half-life in cells of 2 h; this allows prediction that a little more than 10% of the GFP is remaining in the cell 6 h after initiation of GFP expression. We used these data to query the relationship between GFP expression and cell morphology. The studies that have provided evidence for the role of integrin-ECM interactions in the modulation of TN-C promoter activity [8-10], and have reported apparent correlations between cell spreading, actin assembly, stress fiber formation and FAK activation with TN-C promoter activity [8-12] have largely utilized Western blotting, DNA analysis, and other techniques that provide data on the average response of the population. When fluorescence microscopy has been used, it has involved a limited number of cells. Similarly in this study, we observed that cell populations that exhibit larger average cell area in response to the extracellular matrix also exhibit higher average TN-C promoter activity. Interestingly, cell-by-cell analysis, which allows us to examine the correlation between TN-C promoter activity and cell morphology on a per-cell basis, reports only at best a weak correlation between TN-C promoter activity and morphology within individual cells.

The thin film technologies used here provide the means of producing an ECM that can be quantitatively characterized and systematically modified to vary the characteristics of the ECM presented to cells. The different ECM surfaces employed in this study vary in chemical moieties available for integrin engagement (collagen versus fibronectin) and in the density of large collagen fibrils (resulting from low and high concentrations of collagen). The collagen surface preparations used here provide the same chemical recognition sites to cells as has been shown previously by anti-beta 1 integrin blocking studies [44], yet result in different cellular phenotypes. We hypothesize that these collagen surfaces provide different mechanical signals to the cells by virtue of the presence or absence of large collagen fibrils. They also vary in their topography. We are currently expanding the use of thin films to further our understanding of the separate roles that topography and mechanical properties play in cellular response.

Another advantage of the use of thin films of extracellular matrix proteins is that they facilitate unbiased automated fluorescence microscopy data collection and analysis. The three dimensional homogeneity of the thin film and the low level of scattered light makes it easier to automate the collection of data from large numbers of cells. Cell by cell analysis permits direct observation of multiple parameters within individual cells, allowing statistically relevant cell by cell correlations to be determined. Flow cytometry can, of course, also be used to collect multiparametric data from a statistically significant numbers of individual cells. However, flow cytometry requires dissociation of cells from their matrix, eliminating the ability to measure cell-substrate interactions and cell spreading, which microscopic analysis with thin film matrices allows. The ability to examine large numbers of adherent cells quantitatively also provides the potential to examine the development of intracellular signaling pathway networks in live cells in the temporal regime.

Our examination of the response of individual fibroblasts within a population reveals that all cells do not respond identically, but that the population displays a range of responses in area of spreading, intensity of F-actin staining and GFP expression. These distribution patterns are highly reproducible but distinctly dependent on the identity of the ECM matrix. A distribution in phenotypic response is observed even though cells are daughters of a single cell clone and the thin film surfaces on which they are seeded are initially highly homogeneous as verified by AFM, ellipsometry, and light microscopy. While it not the immediate focus of this report, our observations beg the question of how the distributions of responses arise. Preliminary data indicate that the observed distributions do not arise simply as a result of cells in the population being in different stages in the cell cycle. Flow cytometry analysis of propidium iodide stained cells showed that for cells kept for 16 h in medium containing 0.1% serum, 80% of the cells were in the G0/G1 phase, compared to cells left in medium containing 10% serum, in which case 62% of the population were in G0/G1. Yet when plated on thin films of collagen, serum starved cells showed nearly identical distributions in cell area and GFP expression as cells that were not serum starved [see Additional File 1]. Spontaneous instabilities in the cellular genome due to extensive passage does not appear to be a factor, since the distributions are observed in very early passage cells as well as in cells examined at later passages. For the results shown in Figures 2, 4, 5 and 6, the cells were in passage number 12. The cell cycle experiment shown in Additional File 1 was conducted with cells in passage number 29. We have observed that when these cells are kept in continuous culture for as many as 86 passages there is a shift in the distribution of areas relative to the phenotype of younger cells; however, the population continues to display a similar shape in distribution of cell areas.

The observed distributions thus appear to be unique reproducible signatures of the cellular response to the environment and allow statistical evaluation of population heterogeneity. The distribution may reflect statistical fluctuations in intracellular reactions. The effect that stochastic fluctuations in intracellular reactions can have on phenotype has been demonstrated, for example, by Pedraza and van Oudenaarden [45]. These investigators treated the distribution of responses within a population of E. coli cells to model fluctuations in gene expression and determine the effect of fluctuations in one gene on expression of other linked or independent genes. Using a designed gene network, they demonstrated the large effects that global fluctuations can have on gene expression, including the appearance of increases, step functions and decreases in gene expression as a function of upstream noise. In a complex network such as that which controls TN-C promoter activity, it is perhaps not surprising that correlations between TN-C promoter activity and other cellular parameters might be observed on the average, but not in each cell. For a complex eukaryotic system such as TN-C reporter activity in fibroblasts, such noise could be the result of fluctuations in enzymatic reactions leading to activation of the TN-C promoter effectors, or the net response to a combination of promoter effectors acting at different sites on the TN-C promoter. The TN-C promoter is composed of discrete response elements for ECM, stress, and serum, all of which likely play a role in TN-C promoter expression in this study [5-7].

The 24 h time point examined in this experiment is a 'snap-shot' of cell response to the surfaces. Ideally, full characterization of the cellular response would involve quantifying the cell area and GFP intensity in live cells at many time points over an extended period of time. This is experimentally challenging, but is something our lab is currently working on. Non-quantitative examination of live cells over time indicates that after the first few hours cells reach their fully spread condition, and the variation in the extent of spreading of each cell after that time is small relative to the range of variation seen over the entire population; this appears to be true for GFP expression as well [see Additional File 2]. It can be seen that over a 6 hour time frame, each cell exhibits changes in fluorescence intensity and cell area. However, by watching the least spread cells it is apparent that they never become large; weak GFP expressers never become bright; the most spread cells never become as small as the least spread cells; and the bright cells never return to the level of autofluorescence. Nevertheless, the population always exhibits very small cells, very large cells, very bright cells and very dim cells, and intermediate phenotypes. These preliminary data suggest that our 24 h 'snap-shot' is relevant to the response of the population at any time after spreading. It also suggests that the range of variations that occur over the entire population is not due to temporal fluctuations experienced by any individual cell. Thus the distributions reflect a population parameter, and are not representative of the behavior of an individual cell.

## Conclusion

The thin film ECM protein surfaces used in this study possess excellent optical properties for microscopy. This makes it possible to more accurately measure the response of cells on Type 1 collagen than is possible with collagen gels. The use of thin films of ECM proteins provides a high degree of control over surface reproducibility and homogeneity and increases the likelihood that each cell experiences the same microenvironment. Combined with appropriate methodologies for fixing and staining cells, quantitative determination of intracellular fluorescence can be determined on large numbers of cells in an automated fashion. Our data show quantitatively the range of responses that cells display in area of spreading, intensity of F-actin staining, and GFP-TN-C promoter expression. The highly reproducible distributions in phenotypic response occur even though all cells are derived from a single clone and are exposed to a highly homogeneous ECM environment, suggesting that the distributions may be the result of stochastic noise in intracellular processes.

In this study, we observed that cell populations that exhibit larger average cell area in response to the matrix also exhibit higher average TN-C promoter activity. Interestingly, cell-by-cell analysis indicates at best a weak correlation between TN-C promoter activity and morphology within individual cells. The quantitative approaches described here will make possible the further analysis of stochastic influences on phenotype under various ECM conditions and better enable delineation of causal relationships between intracellular parameters.

## Methods^1^

All reagents were purchased from Sigma (Sigma, St. Louis, MO) unless indicated otherwise.

### Preparation of collagen surfaces

The preparation of millimeter thick hydrogels of native fibrillar collagen and thin films of collagen on gold coated coverslips has been described previously [18,27]. Briefly, purified type I collagen was purchased as a solution of acid-stabilized monomer (Vitrogen; Cohesion Technologies, Inc., Palo Alto, CA). The collagen solution (0.8 mL, ≈3 mg/mL, 4°C) was neutralized with 0.1 mL of 10× Dulbecco's phosphate buffered saline (DPBS) (4°C) and 0.1 mL of NaOH (0.1 M, 4°C), and was kept at 4°C to minimize polymerization. To generate the fibrillar gels gaskets (≈16 mm inside diameter, 22 mm outside diameter) were cut from paper labels (Fasson label material, Avery Dennison, Brea, CA) and adhered onto cleaned coverslips before sterilization with 70% ethanol. Neutralized native collagen solution (100 μL) was applied to the dried coverslip making sure that solution fully contacted the paper gaskets. The samples were placed at 37°C overnight to initiate fibrillogenesis. The native collagen hydrogels were carefully rinsed and stored in DPBS at 4°C until they were used.

To prepare thin films for collagen and fibronectin, coverslips were coated with a 5 nm layer of chromium and a 15 nm – 20 nm layer of gold by magnetron sputtering. The semi-transparent gold-coated coverslips were immersed in 0.5 mM 1-hexadecanethiol (Aldrich, Milwaukee, WI) in ethanol for at least 8 hours before being rinsed with ethanol and dried with filtered N_2_. Thin films of fibrillar collagen were generated by adding neutralized solutions of native collagen solution (4°C, ≈0.4 mg/mL) to the alkanethiol-treated gold-coated coverslips and incubating overnight at 37°C. After incubation, the samples were slowly lifted out of the gelled collagen solutions, and rinsed with a stream of DPBS and then water from sterile Teflon squirt bottles (Nalgene Nunc, Rochester, NY). Once all loosely adhered gel was removed, the samples were dried under a stream of filtered N_2 _and immediately placed back into a DPBS solution. Thin films were also produced from a lower solution concentration of collagen by immersing alkanethiol-treated gold-coated glass coverslips into a solution of ≈0.025 mg/mL collagen in DPBS. The samples were incubated at 37°C for at least 12 hours, rinsed in DPBS and water, dried under a stream of filtered N_2 _and stored in DPBS at 4°C before they were used with cells. Ellipsometry studies of the thin films of collagen show that the preparations generated from the 0.4 mg/mL native collagen solution have an average thickness of 40 nm while those prepared from the lower solution of collagen (0.025 mg/mL) are 6 nm on average with a standard deviation of film thickness of less than 10% for both preparations. The homogeneity of surface coverage, evaluated by comparing the thickness of several areas on each sample by ellipsometry indicated that the standard deviation across the samples was less than 4% [27]. To produce thin films of fibronectin, alkanethiol-treated gold-coated glass coverslips were incubated in the presence of 25 μg/mL solution of bovine fibronectin for at least 5 hours at 4°C, rinsed in DPBS and water, dried under a stream of filtered N_2 _and stored in DPBS at 4°C. Ellipsometry indicates that the fibronectin layer has a thickness of 4.5 ± 0.5 nm. Prior to seeding cells on the ECM substrates, the thin films and thicker hydrogels were conditioned for a minimum of 30 minutes with cell culture medium containing 5% (v/v) fetal bovine serum (FBS; Gibco Invitrogen, Carlsbad, CA).

### Cell culture

NIH3T3 fibroblasts (ATCC, Manassas, VA), were maintained in Dulbecco's Modified Eagles Medium (DMEM; Mediatech, Herndon, VA) supplemented with nonessential amino acids, glutamine, penicillin (100 units/mL), streptomycin (100 μg/mL), 10% by volume FBS and 25 mM HEPES, and maintained in a humidified 5% CO_2 _balanced-air atmosphere at 37°C. Sub-confluent cultures were switched to supplemented DMEM containing 5% (v/v) FBS 24 hours prior to an experiment. Cells were removed from tissue culture polystyrene flasks by trypsinization, washed with DMEM/5% FBS, centrifuged for 5 minutes at 200 g and plated in DMEM/5% FBS onto the ECM preparations at a density of 2000 cells/cm^2^. Care was taken to ensure the seeding density was homogeneous over the surface of the substrates. This level of serum of 5% was chosen due to the results of preliminary experiments to examine the relative strength of the serum response element of the TN promoter compared to the biomechanical force and matrix response elements. We chose 5% serum to assure that cell response to the matrix was not overshadowed by the response to serum.

### GFP reporter construct

The TN-1/pEGFP construct containing a 4.1 kilobase fragment of the TN-C promoter was generously provided by Dr. Peter L. Jones (University of Pennsylvania) [37]. The sequences encoding enhanced GFP were removed from this construct using restriction enzymes Sal I and Not I followed by gel purification. A fragment of DNA encoding a degenerate variant of enhanced GFP, with a half-life of 2 hours as reported by the manufacturer, was removed from the vector pd2EGFP-1 (Clontech Laboratories, Palo Alto, CA) using similar restriction enzymes, was gel purified and the resulting 860 base pair fragment was ligated into to the TN-C promoter generating TN-1/pd2EGFP-1. NIH3T3 cells were transfected with the degenerate enhanced GFP-TN-C promoter based reporter construct using Lipofectamine reagent (Invitrogen, Carlsbad, CA) as per manufacturer's recommendation. Briefly, 1.5 × 10^6 ^cells were plated into a 6 well plate 16 hours prior to the transfection in medium lacking antibiotics. A mixture containing 1.5 μg DNA and 8 μL Lipofectamine was added dropwise to cells. Following a 6 hour incubation, fresh medium containing 10% FBS was added to the cells. Cells were maintained in selection medium containing G418/Geneticin (Gibco Invitrogen, Carlsbad, CA) (400 ug/mL) for fourteen days. Two rounds of limited dilution cloning were used to assure that the resulting cell line was derived from a single cell. This clonal population was used for all reporter based experiments. Passage number was designated from the time of isolation of the final subclone. The clone used for this study has been cryopreserved at low passage numbers to allow us to use relatively low passage number cells for replicate experiments at different times. In the course of this study, we have also begun to examine how passage number and cryopreservation quantitatively influence cell response. For the data shown in Figures 2, 4, 5 and 6, cells were used at passage number 12. For the data shown in Additional File 1, the cells were used at passage number 29.

### Cell fixation and staining for automated data analysis

Substrates were removed from the incubator after 24 hours, rinsed with Hanks Balanced Salt Solution (HBSS; ICN Biomedicals, Costa Mesa, CA) supplemented with 10 mM HEPES and fixed for 24 hours at room temperature in 100 mM PIPES, 1 mM EGTA, 4% PEG [46] containing 100 ug/mL 3-malemido-benzoic acid-NHS ester (MBS, Sigma) as the cross-linker [47]. Cells were permeabilized in 0.05% (v/v) Triton X-100 in DPBS, rinsed in DPBS, and incubated with DPBS containing Texas Red-C2-Maleimide (1 ng/mL) as a general stain and 0.05% (v/v) 4',6-diamidino-2-phenylindole (DAPI, Sigma) as a nuclear counter stain. After 2 hours at room temperature, 1% Bovine Serum Albumin (BSA) was added to quench the conjugation reaction. The substrates were then rinsed with DPBS and were mounted onto thin glass slides with 9:1 glycerol:Tris, pH 8.0. In separate control experiments, we have determined that this fixation procedure results in the retention of >90% of the original GFP of the cells (Elliott et al., in preparation). The fixed and stained cells were examined by phase contrast and fluorescence microscopy using a 10× objective on an inverted microscope (Zeiss Axiovert S100TV, Thornwood, NJ) outfitted with a computer controlled stage (LEP, Hawthorne, NY), an excitation filter wheel (LEP, Hawthorne, NY), and a CCD camera (CoolSnap fx, Roper Scientific Photometrics, Tucson, AZ). Hardware operation, and image digitization and analysis were under software control (ISee Imaging, Cary, NC) [18,27]. A modular software routine controlled automated movement of the stage, auto-focusing, and collection of data from 50 to100 independent fields (of 870 μm × 690 μm in size) of cells per sample. Auto-focusing was performed on each field while examining the Texas Red fluorescence. The computer-controlled routine involves sequential steps of moving the objective lens relative to the sample to find the objective position that results in maximum variance in intensities within the field. For each field, cellular fluorescence from Texas Red, FITC or GFP, and DAPI were collected by automated switching of the appropriate excitation and emission filters and passing the emitted light through a multipass beam splitter (set# 84000; Chroma Technology Inc., Brattleboro, VT). The area of each individual cell, and the numbers of cells in each field were determined with image analysis software (ISee Imaging, Cary, NC) as previously described [18,27]. Briefly, appropriate thresholding criteria allowed cell areas, as determined by areas containing Texas Red fluorescence, to be accurately segmented from the non-fluorescent non-cell areas. Fluorescence background around each cell in the GFP and FITC phalloidin stained images was determined by measuring the average background intensity in a defined region just outside the cell area mask described above. The defined region around each cell was generated by systematically enlarging (dilating) the original cell mask The number of nuclei, and therefore the number of cells, was determined from the corresponding images collected with the DAPI filter. Total intensity for the fluorophore in the images was calculated as follows: mean intensity outside the cell was subtracted from the mean intensity within the cell, and the result was multiplied by the cell area. It is likely that each entire cell is sampled because, in addition to auto-focusing, the objective lens used (10× magnification and 2.5 numerical aperture) has a depth of field of 8.5μm, which is on the order of the full thickness of the fibroblasts. Images were collected by moving the stage automatically in 1 mm steps. Morphology and fluorescence intensity were determined for at least 150 individual cells on each substrate.

### Immunofluorescence and phalloidin staining

For immunostaining of actin, paxillin, and phosphorylated FAK Y^397^, cells were fixed in 4% formaldehyde, permeabilized with 0.1% (v/v) Triton X-100 in DPBS (5 minutes), blocked with DPBS containing 30 mg/mL BSA (blocking solutions) and 50 mg/mL donkey or goat serum at room temperature (1 hour). Appropriate samples were stained overnight at 4°C with 5 μg/mL rabbit anti-phospho FAK Y^397 ^(Biosource, Camarilo, CA) or 20 μg/mL anti-paxillin (Upstate Biotechnology, Lake Placid, NY), in blocking solution. The samples were rinsed multiple times in DPBS and incubated with 10 μg/mL rhodamine-labeled donkey anti-rabbit antibody (Chemicon, Temecula, CA) or Alexa 556 goat anti-mouse as appropriate, in blocking solution at room temperature (1 hour). For filamentous actin (F-actin) staining, cells fixed and permeabilized as above were blocked with DPBS containing 30 mg/mL BSA (30 minutes), stained with Alexa 488 phalloidin (Molecular Probes, Eugene OR) in blocking solution (250 nM, 1 hour), and rinsed with blocking solution. The immunostained and phalloidin-stained samples were rinsed extensively with DPBS and samples were mounted on slides in Tris buffered saline containing 90% glycerol, 2.5 mg/mL DABCO and 0.05 μg/mL DAPI. Cells were imaged with phase and fluorescence microscopy using the appropriate filter sets.

### Statistical analysis

Differences between mean values of GFP intensities and cell area for cells on the various surfaces were tested for statistical significance using a one way analysis of variance test in Sigma Stat software (SPSS Inc. Chicago, IL) followed by a Student-Newman-Keuls post hoc test for pair-wise comparison of cellular behavior (area or GFP intensity) on each substrate. A calculated p value of < 0.05 was considered significant in these comparisons.

The correlation between two variables was tested for statistical significance using Spearman rank order correlation test in Sigma Stat software (SPSS Inc. Chicago, IL). The Spearman correlation measures the strength of association between pairs of variables which cannot be described as having a normal distribution with constant variance, which is the case for the cell area data, the phalloidin data and the GFP intensity data The correlation coefficient, r is a number that varies between -1 and +1 with a correlation of +1 indicating a perfect positive relationship between the two variables, with both variables always increasing together. A P value (the probability of being incorrect in concluding that there is a true association between the variables) was calculated and the lower the P value the greater the probability that there is a direct relationship between the two variables. P < 0.05 was considered significant in these comparisons.

## Abbreviations

ECM, extracellular matrix; TN-C, tenascin-C; GFP, green fluorescent protein

## Authors' contributions

KJL: design of cell biology experiments, data collection and analysis; JTE: protocols for surface fabrication and microscopic analysis; AT: experimental set-up and cell staining; DM: Live cell microscopy; ALP: experimental design, data reduction and analysis.

## Note

Indication of specific manufacturers and products is for clarity only and does not constitute endorsement by NIST.

## Supplementary Material

Additional File 1 **Effect of serum starvation on cell cycle and cell response**. A. Flow cytometry of propidium iodide stained cells kept for 16 h in complete medium (left) or in medium with 0.1% serum. The region indicated as 'C' corresponds to G0/G1, 'D' to S and 'E' to G2/M phase of the cell cycle. B. Cell area for cells on thin films of fibrillar collagen (■,□) and thin films prepared from the lower concentration of collagen (●,○). Open circles represent cells that have been serum starved. C. GFP expression on thin films of fibrillar collagen (high) and lower concentration collagen (low). Hatched bars represent cells that have been serum starved. Click here for file

Additional File 2 **Live NIH3T3 TN-1/pd2EGFP-1 cells on fibronectin films**. Cells were kept at 37°C in an incubator chamber on the microscope stage. Images were taken after cells were fully spread (after at least 2 hours after seeding) at 5 min intervals for a total time of 6 h. Three cells are outlined in several frames in the sequence for ease of viewing. Click here for file
