# Supplementary material for: Thin films of Type 1 collagen for cell by cell analysis of morphology and tenascin-C promoter activity
Source: BMC Biotechnol. 2006 Mar 6;6:14. doi: 10.1186/1472-6750-6-14 (PMC1523190; doi:10.1186/1472-6750-6-14)
Supplement: Additional File 1 — Effect of serum starvation on cell cycle and cell response. A. Flow cytometry of propidium iodide stained cells kept for 16 h in complete medium (left) or in medium with 0.1% serum. The region indicated as 'C' corresponds to G0/G1, 'D' to S and 'E' to G2/M phase of the cell cycle. B. Cell area for cells on thin films of fibrillar collagen (■,□) and thin films prepared from the lower concentration of collagen (●,○). Open circles represent cells that have been serum starved. C. GFP expression on thin films of fibrillar collagen (high) and lower concentration collagen (low). Hatched bars represent cells that have been serum starved. [file 1472-6750-6-14-S1.ppt]

## Slide 1
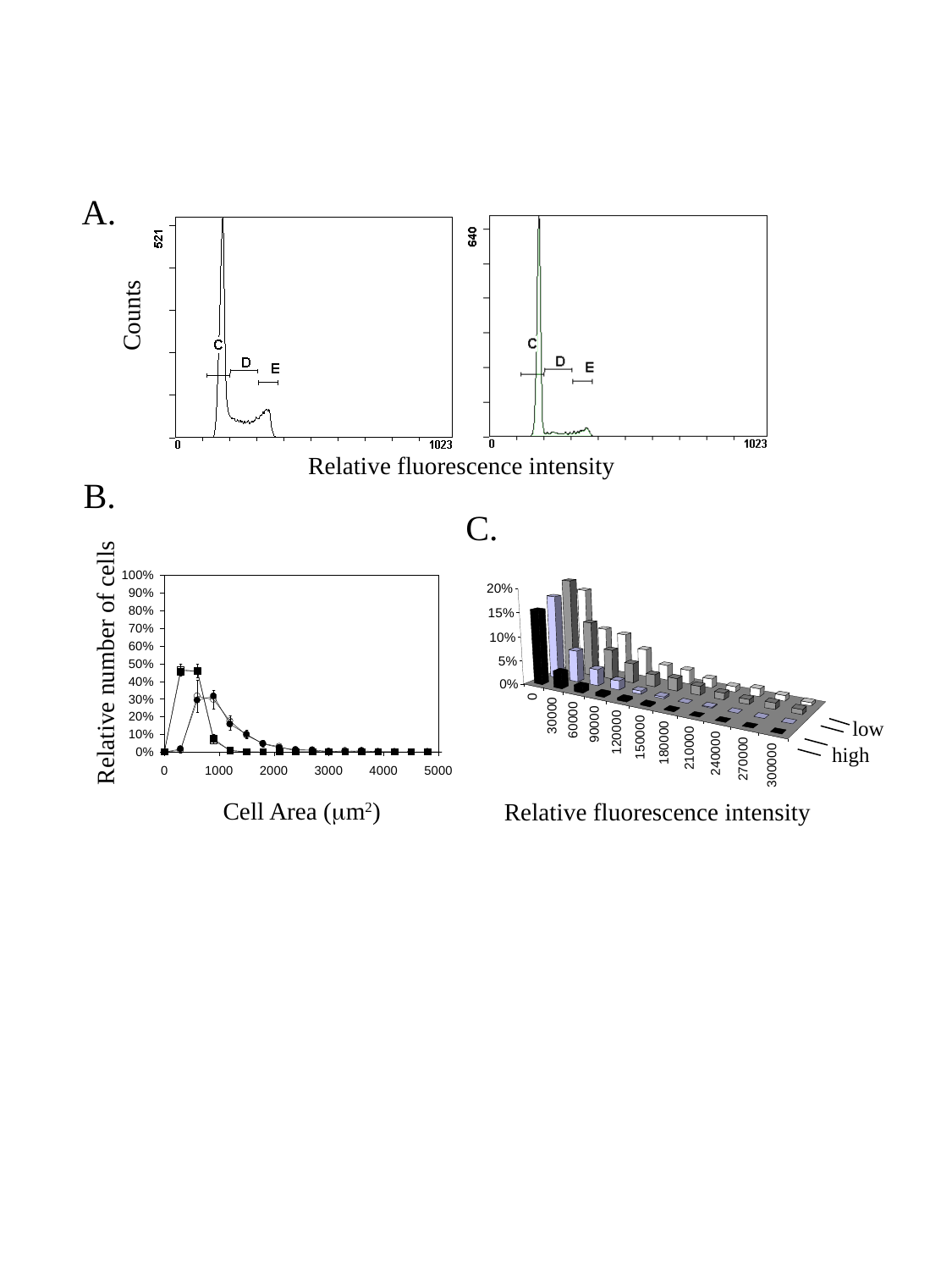

A.
Counts
Relative fluorescence intensity
B.
C.
Cell Area (μm2)
Relative number of cells
low
high
Relative fluorescence intensity
